# *Bacillus subtilis* and *Bacillus licheniformis* reduce faecal protein catabolites concentration and odour in dogs

**DOI:** 10.1186/s12917-020-02321-7

**Published:** 2020-04-19

**Authors:** Tais Silvino Bastos, Daniele Cristina de Lima, Camilla Mariane Menezes Souza, Alex Maiorka, Simone Gisele de Oliveira, Letícia Cardoso Bittencourt, Ananda Portella Félix

**Affiliations:** 1 Department of Animal Science, Federal University of Paraná, Curitiba, 80035-050 Brazil; 2 DSM, São Paulo, SP Brazil

**Keywords:** Biogenic amines, Direct-fed microbials, Faecal consistency, Intestinal functionality

## Abstract

**Background:**

Direct-fed microbials (DFM), such as *Bacillus subtilis* and *Bacillus licheniformis*, may improve gut functionality of the host by favouring non-pathogenic bacteria and reducing the formation of putrefactive compounds. The aim of this study was to assess the nutrient digestibility, faecal characteristics and intestinal-fermentation products in dogs fed diets with *Bacillus subtilis* and *Bacillus licheniformis*. Sixteen dogs were randomly divided into two groups. Every eight dogs were fed with the control diet or the diet with the addition of 62.5 g of DFM (*B. subtilis* and *B. licheniformis*)/ton. Diets were provided throughout a 20-day adaptation period, followed by 5 days of total faecal collection. Nutrient digestibility and the metabolisable energy of the diets, plus the dogs’ faecal characteristics and intestinal fermentation products were assessed.

**Results:**

There were no differences in nutrient digestibility (*P >* 0.05). However, DFM supplementation improved faecal score and resulted in less fetid faeces (*P <* 0.001). DFM inclusion reduced (*P <* 0.05) the biogenic amines concentration: putrescine, spermidine and cadaverine, besides the concentration of phenols and quinoline.

**Conclusions:**

The use of *B. subtillis* and *B. licheniformis* as DFM reduce the concentration of nitrogen fermentation products in faeces and faecal odour, but the digestibility of nutrients is not altered in dogs.

## Background

Many complete dog foods have a high protein content in their formulations, which can result in high concentrations of undigested nitrogen compounds in the large intestine, leading to the formation of putrefactive compounds. These compounds include ammonia, biogenic amines, branched-chain fatty acids, sulphidric gas, phenols and indoles [1]. Some of these catabolites can negatively affect intestinal functionality, contributing to inflammatory processes, such as colitis and colon carcinogenesis, as well as the worsening of dogs’ faecal odour [1, 2]. Furthermore, the increase in the non-digestible-protein flow that reaches the colon provides fermentation substrates to organisms with pathogenic potential, such as *Clostridium*, *Salmonella* and *Escherichia* species [3, 4], and can contribute to dysbiosis, an imbalance of the intestinal microbiota.

Thus, the search for nutritional strategies, such as the use of direct-fed microbials (DFM) to improve dogs’ intestinal functionality and faecal quality is extremely relevant. DFM has been defined by the US Food and Drug Administration as the feed product containing the source of naturally-existing live microbes, like some bacteria of the *Bacillus* genus. When supplemented in the diet, these microorganisms may favour non-pathogenic bacteria [5, 6].

Bacteria of the *Bacillus* genus, such as the facultative anaerobic species *B. subtillis* and *B. licheniformis,* have the advantage of sporulation. This characteristic makes them more viable in food and resistant to acidic gastric pH [6–8]. *B. subtilis* and *B. licheniformis* are commonly found as spores in the soil. The spores are dehydrated and when exposed to appropriate nutrients and moisture will germinate in the small intestine, resuming their cell vegetative growth [9]. After being excreted, these organisms can sporulate again in faeces [7, 10].

Studies have shown that *B. subtilis* and *B. licheniformis* can improve faecal odour and reduce gas formation in the intestine of dogs [11], prevent necrotic enteritis in broilers [12], and reduce diarrhoea in piglets [13]. Since they are viable after excretion, these organisms degrade organic matter in the faeces, reduce ammonia production [4] and potentially reduce faecal odour. Given the above, the aim of the present study was to evaluate diet digestibility, faecal characteristics and intestinal fermentation products in dogs supplemented with DFM *Bacillus subtilis* and *Bacillus licheniformis*.

## Results

All dogs remained healthy throughout the experiment. No episodes of vomiting or diarrhoea were observed. No differences in DM intake (Control = 201.2 + 4.33 g/day and DFM = 204.1 + 5.34 g/day) and body weight (Control =10.2 + 1.04 kg and DFM = 10.5 + 1.08 kg) were observed between treatments (*P* > 0.05).

**Fig. 1:**
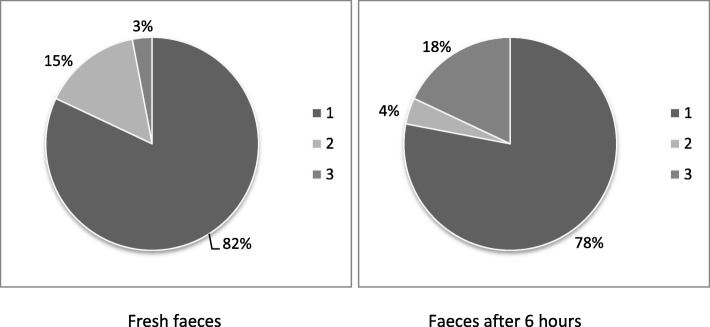
Points frequency of faecal odour scores attributed by evaluators (*n* = 50). 1 = less fetid odour; 2 = same odour and 3 = more fetid odour, as compared to faeces of dogs fed the control diet

The inclusion of DFM with *Bacillus subtilis* and *Bacillus licheniformis* in extruded dog diets did not alter (*P >* 0.05) the nutrient apparent total tract digestibility (ATTD) coefficients and diet metabolisable energy (ME) (Table 1), faecal DM, ammonia, faecal pH, and sialic acid (Table 2), as well as the SCFA and BCFA concentrations in the faeces (Table 3). The faecal score, however, was increased and the inclusion of DFM resulted in less fetid faeces both in fresh samples and 6 h after defecation (*P <* 0.05, Table 4 and Fig. 1). According to 82% of the evaluators, fresh faeces of dogs consuming the DFM diet were less fetid than those from animals consuming the control diet. Similarly, 78% of evaluators considered that 6 h after defecation the faeces of dogs consuming DFM were less fetid than those in the control group (*P <* 0.05, Fig. 1). The concentration of biogenic amines: *putrescine*, spermidine and cadaverine, as well as phenols and quinoline were reduced (*P <* 0.05) with the inclusion of DFM (Tables 5 and 6).

**Table 1:** Diet ATTD and ME means with or without DFM (*n* = 8)

Item	Control	DFM	SEM	*P-value*
ATTD (%)				
Dry matter	79.1	78.8	0.31	0.739
Organic matter	84.1	83.8	0.25	0.754
Crude protein	79.9	78.8	0.56	0.281
Ether extract	86.5	85.6	0.42	0.297
Ash	43.9	44.2	0.45	0.921
Nitrogen-free extract	92.9	92.1	0.67	0.892
ME (kcal/kg)	4034.9	4062.4	10.49	0.199

*ATTD* Apparent total tract digestibility, *ME* Metabolisable energy

*DFM* Direct-fed microbials (62.5 mg/kg of diet of a mixture of 3.66 × 10^7^ cfu/kg *Bacillus subtilis* and 3.66 × 10^7^ cfu/kg *Bacillus licheniformis*)

**Table 2:** Faecal characteristics means of dogs fed diets with or without DFM (n = 8)

Item	Control	DFM	SEM	*P-value*
Fresh faeces pH	6.32	6.50	0.058	0.128
After 6 h pH	6.32	6.31	0.035	0.933
Fresh faeces moisture (%)	70.04	69.12	0.563	0.434
After 6 h moisture (%)	67.39	67.36	0.707	0.981
Fresh faeces ammonia (g/kg)	0.52	0.50	0.001	0.834
After 6 h ammonia (g/kg)	0.73	0.61	0.001	0.897
Faecal production	0.13	0.12	0.005	0.379
Sialic acid (μmol/g)	2.46	2.47	0.04	0.943

*DFM* Direct-fed microbials (62.5 mg/kg of diet of a mixture of 3.66 × 10^7^ cfu/kg *Bacillus subtilis* and 3.66 × 10^7^ cfu/kg *Bacillus licheniformis*)

**Table 3:** SCFA and BCFA means in faeces of dogs fed diets with or without DFM (n = 8)

Item	Control	DFM	SEM	*P-value*
SCFA (μmol/g)				
Acetic	31.21	33.79	1.118	0.263
Propionic	27.66	27.16	1.205	0.845
Butyric	4.78	3.94	0.316	0.193
Total SCFA	64.33	64.89	1.899	0.887
BCFA (μmol/g)				
Isobutyric	0.79	0.91	0.035	0.090
Isovaleric	0.78	0.67	0.044	0.198
Valeric	0.29	0.31	0.011	0.415
Total BCFA	1.94	1.87	0.065	0.640

*SCFA* short chain fatty acids, *BCFA* branched chain fatty acids

*DFM* Direct-fed microbials (62.5 mg/kg of diet of a mixture of 3.66 × 10^7^ cfu/kg *Bacillus subtilis* and 3.66 × 10^7^ cfu/kg *Bacillus licheniformis*)

**Table 4:** Faecal score, odour medians and interquartiles of dogs fed diets with or without DFM

Item	Control	DFM	*P-value*
Faecal score	4 (3/4)	4 (4/4)	< 0.001
Odour fresh faeces	2 (2.0/2.0)	1 (1.0/1.0)	< 0.001
Odour after 6 h	2 (2.0/2.0)	1 (1.0/1.0)	< 0.001

*Faecal score* (n = 8) 1 (liquid stools) to 5 (dry stools); *faecal odour* (n = 50) 1 (less fetid than control) 2 (same as control) and 3 (more fetid than control)

*DFM* (Direct-fed microbials) (62.5 mg/kg of diet of a mixture of 3.66 × 10^7^ cfu/kg *Bacillus subtilis* and 3.66 × 10^7^ cfu/kg *Bacillus licheniformis*)

**Table 5:** Biogenic amines (mg/kg) means in faeces of dogs fed diets with or without DFM (n = 8)

Item	Control	DFM	SEM	*P-value*
Tiramine	48.36	46.22	10.770	0.443
Putrescine	71.24	47.51	9.321	0.025
Cadaverine	129.87	88.63	18.935	0.050
Histamine	22.93	23.89	5.280	0.448
Serotonin	3.20	2.39	0.381	0.057
Agmatine	0.00	0.00	0.000	1.000
Spermidine	26.77	19.94	2.451	0.015
Phenylethynamine	0.55	0.92	0.253	0.147
Tryptamine	0.51	0.22	0.344	0.272
Total amines	303.42	209.90	38.301	0.031

*DFM* Direct-fed microbials (62.5 mg/kg of diet of a mixture of 3.66 × 10^7^ cfu/kg *Bacillus subtilis* and 3.66 × 10^7^ cfu/kg *Bacillus licheniformis*)

**Table 6:** Mean percentage of peak areas of more abundant volatile organic compounds present in the dogs’ faeces (n = 8)

	Item	Control	DFM	SEM	*P-value*
Fresh faeces	Phenols	37.18	18.96	3.13	< 0.001
	Quinoline	14.57	3.06	3.14	< 0.001
	Indoles	63.32	67.08	2.98	0.548
After 6 h	Phenols	33.48	34.58	2.86	0.855
	Quinoline	7.68	7.33	1.85	0.927
	Indoles	58.83	58.04	3.98	0.929

DFM (Direct-fed microbials) (62.5 mg/kg of diet of a mixture of 3.66 × 10^7^ cfu/kg *Bacillus subtilis* and 3.66 × 10^7^ cfu/kg *Bacillus licheniformis*)

## Discussion

This study demonstrates that dietary DFM supplementation reduced the concentration of protein fermentation compounds in faeces, resulting in lower faecal odour of dogs. However, it did not change diet digestibility.

The absence of DFM effects on diet digestibility components was similar to that observed by Biourge [14] and Pasupathy [15]. The authors found no difference in the digestibility of dog diets containing 7.5 *×* 10^6^*cfu of Bacillus CIP 5832* and 2 × 10^7^ cfu of *Lactobacillus acidophilus*.

Besides nutrient digestibility, faecal characteristics should also be taken into consideration in dog-food evaluations. Faecal characteristics can be a reflex of intestinal functionality and have become more relevant for pet owners that look for dog foods that reduce faecal odour and improve faecal consistency. When compared to the control diet, the effect of DFM was reflected by an improvement in faecal consistency. These results were also reported by Félix et al. [6], using a 0.01% supplement of *Bacillus subtilis* (C-3102) and Paap et al. [11], using 0.5 g/100 g *Bacillus subtilis* (C-3102) in diets for dogs. Similarly, when supplement in diet *Bacillus subtilis* and *Bacillus licheniformis*, Alexopoulos et al. [13] reported a diarrhoea reduction in piglets.

The maintenance of intestinal eubiosis plus SCFA production in the colon can explain the increase in faecal consistency [16] with the use of probiotics and DFM in the diet. SCFA production by intestinal microorganisms and their absorption by the colonocytes stimulate water and electrolyte absorption, as well as increase the absorption rate of sodium, responsible for most of the water absorbed in the intestinal lumen [17]. Despite this, faecal SCFA concentration was not altered in the present study with the use of DFM. This may be due to the rapid absorption rate of SCFA by the intestinal mucosa [1, 18].

Likewise, no changes were observed in dogs’ faecal pH, in agreement with what has been reported by Swanson et al. [1], Félix et al. [6], and Stercova et al. [19]. On the other hand, Feliciano et al. [5] observed a reduction in faecal pH of dogs fed a diet containing *Lactobacillus* spp. The absence of faecal pH alterations of dogs fed with *Bacillus* spp. can be due to the limited ability of the *Bacillus* genus species to produce lactic acid when compared to *Lactobacillus* [6].

Amino acid fermentation catabolites are considered to be the main odoriferous components of faeces and can have negative influences on intestinal functionality due to their toxicity and their favouring the survival of bacteria with pathogenic potential [20–22]. Several putrefactive compounds can be produced from the fermentation of undigested amino acids by deamination, deamination-decarboxylation or carboxylation [23], with ammonia, biogenic amines, BCFA, indoles, phenols, and volatile compounds containing sulphur being the major groups [22, 23].

In the present study, DFM supplementation decreased the concentration of potentially toxic putrefactive compounds for the intestinal mucosa when in high concentrations. The reduced compounds were: putrescine, spermidine, cadaverine, phenols, and quinoline, which resulted in faeces with less odour, both when fresh and 6 h after defecation.

Besides these results, the faecal ammonia concentration was not reduced in dogs fed the diet containing DFM. Likewise, other studies did not report a same pattern among the increase or decrease of all protein catabolites [1, 3, 24]. This may be explained by the different nitrogen substrates that generate these catabolites and their different utilization rates by the gut microbiota. Ammonia is produced by deamination, while biogenic amines, phenols, and indoles are produced by the decarboxylation of amino acids. The amine putrescine, spermidine and cadaverine are produced from the decarboxylation of ornithine, methionine and lysine, respectively [25]. Phenols and indoles are produced from the fermentation of tyrosine and tryptophan, respectively [26]. Since these mechanisms are mediated by enzymes produced mainly by intestinal bacteria with pathogenic potential, it is possible that DFM influenced the intestinal microbiota, favouring the non-pathogenic bacterial population.

The fact that the odour was still low 6 h after defecation (simulating what usually happens in the home environment) indicates that *B. subtillis* and *B. licheniformis* have a continuous action in faeces after excretion. According to Vainshtein et al. [4], spore-forming *Bacillus* species produce substances that are antagonists to the development of organisms with pathogenic potential (generally proteolytic) and produce enzymes that degrade OM present in the excreta, reducing ammonia production. These effects can occur both in the gut and the faeces. Still according to these authors, *Bacillus-*genus bacteria have shown major results in sanitizing poultry and swine waste.

Another mechanism that may have contributed to the intestinal microbiota balance could be the immunomodulatory action of *B. subtillis* and *B. licheniformis* in the gut [27], reducing the establishment of organisms with pathogenic potential, protecting the villi and the absorption surface against irritating toxins [28], such as biogenic amines, phenols and indoles. In a study with broilers, Knap et al. [12] observed that the use of *Bacillus licheniformis* in the diet helped prevent necrotic enteritis.

Considering the discussion above, dietary supplementation with DFM *B. subtilis* and *B. licheniformis* has potential beneficial effects in gut functionality in dogs. To our knowledge, this was the first study to describe the reduction on biogenic ammines, phenols, and quinoline in faeces of dogs fed DFM. Besides, the reduction of the faecal odour and improvement in the faecal score are very important commercial characteristics, considering the close relation between dogs and their owners. Different from conventional probiotics, these DFM may be more effective in reaching the colon, considering their ability to form spores and resist to environment and gastric pH levels [7, 8]. These characteristics make DFM very interesting for practical applications in commercial dog diets.

Despite these potential benefits, one limitation of the present study was that we did not evaluate the faecal microbiota of dogs. Besides, although many studies report the toxic effects of higher concentrations of nitrogen fermentative products to colonocytes [20–22], we still have a lack of information of which is the limit between the functional and toxic concentrations of these compounds in the gut mucosa of dogs. This is important considering that dogs have greater protein requirements than humans and it was previously reported that the modulation of some polyamines is important to reduce inflammatory processes and cell infiltrations in dogs with inflammatory bowel disease and colonic polyps [29]. Thus, further studies evaluating the effects of DFM supplementation in faecal microbiota and nitrogen fermentative products in dogs are required to better understand their effects on intestinal functionality and the modulation of dogs’ intestinal microbiota.

## Conclusions

The inclusion of 3.66 × 10^7^ cfu/kg of *Bacillus subtilis* feed and 3.66 × 10^7^ cfu/kg of *Bacillus licheniformis* feed in extruded dog diets improves faecal consistency and odour. It also reduces faecal concentration of compounds produced in protein catabolism such as putrescine, spermidine, cadaverine, phenols, and quinoline, demonstrating possible beneficial effects on dog’s intestinal functionality.

## Methods

### Animals and housing

Sixteen adult intact beagle dogs were used (eight males and eight females), with an average body weight of 10.3 + 1.07 kg and 4 years of age. All animals underwent previous clinical and physical examinations, were vaccinated, dewormed, and individually housed in covered brickwork kennels (5 m long × 2 m wide), containing a bed and free access to fresh water. The environment temperature ranged from 16 °C to 28 °C with a 12-h light–dark cycle (light 6 am–6 pm). All animals were brought to the Research laboratory on canine nutrition of the Federal University of Parana (Curitiba, PR, Brazil) from Maiorca Kennel (Colombo, PR, Brazil) when they were 3–4 months old.

During most of the diet adaptation period (until the 16th day) dogs had free supervised access to an outdoor area for 2 h a day. Between days 17–25 the dogs were individually housed at the kennels to allow for faecal collection. All dogs received extra attention and kennel enrichment during this period. The dogs will be donated when they complete 6 years of age. The use of animals for this study was approved by the Ethics Committee on Animal Use from the Agrarian Sciences Sector, Federal University of Paraná, Curitiba, PR, Brazil (012/2019).

### Experimental diets

The same commercial diet for adult dogs was divided into two parts and used in the experimental treatments. One part (eight dogs, four males and four females) was used in the control treatment, with no DFM supplementation, and the other part (eight dogs, four males and four females) was used as the test treatment containing 62.5 mg/kg of a diet with a mixture of *Bacillus subtilis* (3.66 × 10^7^ cfu/kg of the diet) and *Bacillus licheniformis* (3.66 × 10^7^ cfu/kg of the diet) as DFM (PureGro®, DSM, Heerlen Netherlands). The diet had the following composition: poultry viscera meal, meat meal, corn, soybean meal, poultry fat, swine liver hydrolysate, sodium chloride, citric acid, antioxidants (BHT, BHA), propionic acid, vitamin A, vitamin D3, vitamin E, vitamin B1, vitamin B6, vitamin B12, vitamin K3, nicotinic acid, folic acid, biotin, calcium pantothenate, zinc sulfate, calcium iodate, sodium selenite, copper sulfate, iron sulfate, manganese sulfate and zinc oxide. The chemical composition of the experimental diets is shown in (Table 7).

**Table 7:** Analysed chemical composition of the experimental diets (dry matter basis, %)

Item	Control	DFM
Dry matter	91.77	91.51
Crude protein	21.75	21.12
Ether extract in acid hydrolysis	9.22	9.04
Ash	7.02	7.00
Crude fibre	2.23	2.42
Calcium	1.18	1.23
Phosphorus	0.89	0.91

*DFM* Direct-fed microbials (62.5 mg/kg of a diet with a mixture of 3.66 × 10^7^ cfu/kg *Bacillus subtilis* and 3.66 × 10^7^ cfu/kg of *Bacillus licheniformis*)

DFM was diluted in poultry viscera oil and used on top of the test diet. The same amount of oil, without DFM, was used on the control treatment, ensuring that the diets were isonutritive.

### Experimental procedures

The digestibility assay followed the total faeces collection method as recommended by the Association of American Feed Control Official [30]. The diets were provided during a 20-day adaptation period, followed by 5 days of total faeces collection, resulting in a mixture of faeces from each animal.

The food was provided twice a day (8:30 a.m. and 4:00 p.m.), in amounts sufficient to meet the animal’s metabolisable energy (ME) requirement according to the National Research Council [31], where: ME (kcal/day) = 130 x Body weight^0.75^_._ Water was provided ad libitum. The faeces were collected and weighed at least two times per day and stored in individual previously-identified plastic containers, covered and stored in a freezer (− 14 °C) to be analysed later.

At the end of the collection period, the faeces of each replicate were thawed at room temperature and homogenized separately, forming a composite sample from each animal. Faeces were dried in a forced ventilation oven (320-SE, Fanem, São Paulo, Brazil) at 55 °C for 48 h or until reaching constant weight. Diets and faeces were ground to 1.0 mm in a hammer mill (Arthur H. Thomas Co., Philadelphia, PA, USA), using 1.0-mm wire mesh sieves for the bromatological testing (in duplicate and with repetitions when the variation was higher than 5%).

The amounts of dry matter at 105 °C (DM105), crude protein (CP, method 954.01), crude fibre (CF, method 994.13), ether extract in acid hydrolysis (EEAH, method 954.02), and ash (942.05) were determined in both diets and faeces according to the Association of the Official Analytical Chemists [32]. The amount of gross energy (GE) was established using a calorimetric pump (Parr Instrument Co., model 1261, Moline, IL, USA), and organic matter (OM) was calculated by the difference between 100 – Ash. Nitrogen-free extract was calculated as 100 – CP – Ash – CF – EEAH.

Faecal characteristics were assessed at the end of the study by analysing the total amount of dry faecal matter (DMf), faeces production (g faeces/g DM intake/5 days), consistency score and odour, pH, ammonia concentration, short-chain fatty acids (SCFA), branched-chain fatty acids (BCFA), phenols, indoles, sialic acid, and biogenic amines.

Considering that the faecal-consistency scoring system is a subjective evaluation, the sample was always evaluated by the same researcher using a 5-point rating scale: 1 = faeces are soft and have no defined shape; 2 = faeces are soft and poorly formed; 3 = faeces are soft, formed and moist; 4 = faeces are well formed and consistent; 5 = faeces are well formed, hard and dry, according to Carciofi [33].

Faecal odour was evaluated and scored on the 25th day of the experimental period. Faeces from three animals per treatment were randomly collected, homogenized and the same amounts (5.0 g) were placed in plastic containers of the same size and covered with plastic film with holes (same number and size). The containers were classified as: A (control diet) and B (DFM diet), so the participants would not have information about the treatment. The sensorial analysis was performed by 50 evaluators with fresh faeces (up to 30 min after defecation) and 6 hours after defecation, with different people at each point in time. In the evaluation, sample B with DFM was compared to A (control diet) using the following scoring system: 1 = better odour than control (less fetid); 2 = same as control; 3 = worse than control (more fetid).

Faecal pH and ammonia concentrations were analysed in faeces collected up to 15 min after defecation. Faecal pH was measured in a digital pH meter (331, Politeste Instrumentos de Teste Ltda, São Paulo, SP, Brazil) using 3.0 g of fresh faeces diluted in 30 mL of distilled water. The ammonia concentration was determined according to the method described by Brito et al. [34].

Fresh faeces collected up to 15 min after defecation were used to determine SCFA and BCFA. A properly labelled plastic container with a lid was used to weigh 10 g of faeces mixed with 30 mL of 16% formic acid. This mixture was homogenized and stored at 4 °C for 3 to 5 days. Before the analysis, these solutions were centrifuged at 5000 rpm (2 K15 centrifuge, Sigma, Osterodeam Hans, Germany) for 15 min. At the end, the supernatant was separated and centrifuged. Each sample underwent three centrifugations and, at the end of the last one, part of the supernatant was transferred to a properly identified eppendorff for subsequent freezing. Later on, the samples were thawed and centrifuged again at 14000 rpm for 15 min (Rotanta 460 Robotic, Hettich, Tuttlingen, Germany). Faecal SCFA and BCFA were determined by gas chromatography (Shimadzu®, model GC-2014, Kyoto, Japan) using a 30-m long and 0.32-mm wide glass column (Agilent Tecnologias, HP INNO cera-19,091 N, Santa Clara, USA). Nitrogen was used as the carrier gas at a 3.18 mL/min flow rate. Working temperatures were 200 °C at injection, 240 °C in the column (at a 20 °C/min rate), and 250 °C in the flame ionization detector.

Phenols and indoles were analysed by chromatography using a GCMS2010 Plus gas chromatographer (Shimadzu®) coupled to a TQ8040 mass spectrometer with an AC 5000 autosampler and a split-splitless injector. Chromatographic separations were obtained in the SH-Rtx-5MS (30 m × 0.25 mm × 0.25 μm - Shimadzu®) column with a 1.0-mL min^− 1^ flow rate, and helium as the drag gas at a 5.0 rate. The transfer line and ionization source temperatures were maintained at 40 °C and 220 °C, respectively, with the 1-L injection volume in the split mode (1:10 rate). The GC oven temperature was maintained at 220 °C (5 min) with a 40 °C/min^− 1^ increase to 280 °C (5 min). Total analysis time was 31 min and the mass spectrometer operated in the full scan modes (m/z = 40 to 400) and selective ion monitoring (SIM), with electron ionization at 70 eV. GCMSsolution® was the software used in the data analysis.

For the sialic acid determination, faeces were lyophilized (Alpha 1–4 LO plus, Christ, Osterodeam Hans, Germany) and analysed according to the method described by Jourdian et al. [35]. Biogenic amines were analysed according to the method described by Urrego et al. [36] in fresh faeces, collected up to 15 min after defecation.

The DMf, consistency score, faecal odour, pH, ammonia, phenols and indoles were also analysed in the same samples 6 h after defecation. For the analysis performed 6 h after defecation, faeces were maintained at room temperature (average of 24.5 °C, 84% relative air humidity and in the shade for 6 h.)

### Calculations and statistical analyses

Based on the laboratory results, the apparent total tract digestibility (ATTD) coefficients and the diet’s ME were calculated according to the Association of American Feed Control Official [30]:

ATTD% = [(g of nutrient intake – g of nutrient excretion)/g of nutrient intake] × 100.

ME (kcal/g) = {kcal/g GE intake – kcal/g GE faecal excretion – [(g CP intake – g CP.

faecal excretion) × 1.25 kcal/g]} / g of feed intake.

The experiment had a completely randomized design with two treatments, each one with eight replicates, except for faecal odour that had 50 replicates. Each dog was considered an experimental unit. The Shapiro-Wilk test was used to determine normality of the data and the homoscedasticity of variances was analysed by Bartlett’s test. When these assumptions were met, the t-Student’s test was used at a 5% significance level. The non-parametric data were analysed by the Mann-Whitney-Wilcoxon test (*P <* 0.05). The frequency of faecal odour scores was analysed by the chi-square test (*P <* 0.05).

## Data Availability

All data generated or analysed during this study will be available from the corresponding author upon reasonable previous request and with the permission of the DSM.

## Abbreviations

DFM: Direct-fed microbials
ME: Metabolisable energy
CP: Crude protein
EEAH: Ether extract in acid hydrolysis
DM: Dry matter
DMf: Dry faecal matter
GE: Gross energy amount
OM: Organic matter
SCFA: Short-chain fatty acids
BCFA: Branched-chain fatty acids
ATTD: Apparent total tract digestibility coefficients
